# Obesity Risk Assessment Tool for Low-Income Spanish Speaking Immigrant Parents with Young Children: Validity with BMI and Biomarkers of Obesity

**DOI:** 10.3390/nu12113582

**Published:** 2020-11-22

**Authors:** Marilyn S. Townsend, Mical K. Shilts, Louise Lanoue, Christiana Drake, L. Karina Díaz Rios, Dennis M. Styne, Nancy L. Keim, Lenna Ontai

**Affiliations:** 1 Department of Nutrition, University of California, Davis, CA 95616, USA; llanoue@ucdavis.edu; 2 Department of Family and Consumer Sciences, Nutrition, Food & Dietetics Program, California State University Sacramento, Sacramento, CA 95819, USA; shiltsm@csus.edu; 3 Department of Statistics, University of California at Davis, Davis, CA 95616, USA; cmdrake@ucdavis.edu; 4 Division of Agriculture and Natural Resources, Public Health Department, University of California, Merced, CA 95343, USA; kdiazrios@ucmerced.edu; 5 Pediatric Endocrinology, Department of Pediatrics, University of California, Davis Medical Center, Sacramento, CA 95817, USA; dmstyne@ucdavis.edu; 6 USDA Western Human Nutrition Research Center, University of California, Davis, CA 95616, USA; nancy.keim@usda.gov; 7 Department of Human Ecology, University of California, Davis, CA 95616, USA; lontai@ucdavis.edu

**Keywords:** child obesity prevention, evaluation, Hispanic, low-income families, pre-school, risk assessment, validation, Spanish

## Abstract

Children of Hispanic origin bear a high risk of obesity. Child weight gain trajectories are influenced by the family environment, including parent feeding practices. Excessive body fat can result in unhealthful metabolic and lipid profiles and increased risk of metabolic diseases. The objective was to estimate criterion validity of an obesity risk assessment tool targeting Spanish-speaking families of Mexican origin using anthropometric measures and blood values of their young children. A cross-sectional study design with five data collection sessions was conducted over an eight-week period and involved 206 parent/child dyads recruited at Head Start and the Special Supplemental Nutrition Program for Women, Infants and Children in Northern California. Main outcome measures were criterion validity of *Niños Sanos*, a pediatric obesity risk assessment tool, using anthropometric measures and blood biomarkers. *Niños Sanos* scores were inversely related to child BMI-for-age percentiles (*p* = 0.02), waist-for-height ratios (*p* = 0.05) and inversely related to blood biomarkers for the metabolic index (*p* = 0.03) and lipid index (*p* = 0.05) and positively related to anti-inflammatory index (*p* = 0.047). Overall, children with higher *Niños Sanos* scores had more healthful lipid, metabolic and inflammatory profiles, as well as lower BMI-for-age percentiles and waist-to height ratios, providing evidence for the criterion validity of the tool. *Niños Sanos* can be used by child obesity researchers, by counselors and medical professionals during clinic visits as a screening tool and by educators as a tool to set goals for behavior change.

## 1. Introduction

In the U.S., Hispanic children, 2–19 years old, have the highest prevalence of >85th BMI percentile (46%). This rate is far greater than the national average of 35% for children and that measured in non-Hispanic white children of 30% [1]. Of great concern is the sharp increase in obesity rates >95th percentile BMI in 2–5 year-old children, with Hispanics having a higher prevalence across nearly all classes of obesity and all years between 1999 and 2016 [1,2]. Given that early childhood obesity strongly predicts adolescent and young adult obesity [3] and recognizing that this young age may be ideal for intervention, the Institute of Medicine (IOM) and the American Academy of Pediatrics (AAP) recommended the development of assessment tools targeting families’ modifiable environmental and behavioral factors associated with the risk of pediatric obesity [4,5]. Although many factors have been associated with the development of childhood obesity, excessive caloric intake and/or inadequate physical activity remain the main drivers [6]. Moreover, Hispanic children are disproportionate carriers of genetic variants which predispose them to early onset obesity and fatty liver [7,8]. Elevated BMI in children does not always relate to central obesity [9]. Moreover, waist circumference normalized to height has been an indicator of increased cardiovascular risk in children. However, this anthropometric value may not fully capture the different distribution of body fat, particularly the metabolically active visceral fat that releases mediators, i.e., adipokines. These adipokines in blood have been linked to metabolic dysregulation, dyslipidemia and chronic low-grade inflammation [10,11]. These biomarkers have the potential to be more sensitive indicators of metabolic disturbances in response to obesogenic behaviors than anthropometric measures alone; biomarkers inform adipose tissue dysfunction rather than reflect the accumulation of body fat [10]. Children with obesity tend to have higher circulating blood insulin and leptin levels and may develop resistance to the actions of insulin and leptin [12]. As insulin is critical for cellular glucose uptake and to prevent efflux of fat from the adipose tissue, also known as lipolysis inhibition, children with insulin resistance are at risk of developing type 2 diabetes and cardiovascular complications at an earlier age. HOMA-IR (homeostatic model assessment of insulin resistance) and triglycerides to HDLC ratio (TGHDL) are valid clinical surrogates of insulin resistance and are predictors of metabolic syndrome and cardiovascular risks in children [12,13]. In children, high levels of leptin and insulin are often associated with low levels of adiponectin and high levels of resistin, as obesity is a condition that modulates the secretion of both adipokines, which exacerbates insulin resistance [14,15]. Hence, the leptin to adiponectin ratio is a better marker of body fat and of comorbidity risk in children [12,16]. In addition to antagonizing the action of insulin, resistin is an inflammatory molecule that promotes inflammation and inflammatory diseases [17]. In addition, children with overweight or obesity tend to have lower concentrations of IL-10 and IGFBP-1 [12,18,19,20,21], which can predispose them to low grade inflammation often reflected in higher circulating levels of CRP and resistin [22,23]. Longitudinal studies have shown that children with high BMI have increased risk of cardiovascular disease in adulthood [24]. Of greater concern is the observation that dyslipidemia, insulin resistance and inflammation may begin in childhood for children with obesity. As such, biomarkers in complement to anthropometric measures are useful for the validation of behavioral assessment tools to identify the children who are at higher risk of developing obesity-related diseases including metabolic syndrome, cardiovascular problems, and asthma [4,10,25].

Parents influence young children’s growth trajectories by shaping the home environment, controlling food availability and promoting healthy behaviors [26]. As such, parents play a critical role in establishing the lifestyle behaviors that influence obesity risks and are practiced in the home environment. For Hispanic low-income households, parent socioeconomic and education status are critical factors influencing the obesity risk in children [27]. Hispanic participation in food assistance programs has been reported to be lower than among the general eligible population [28]. At the same time, Hispanic households report some of the highest rates of food insecurity [29]. In response to the staggering obesity rates among children, Congress authorized federal programs to include an obesity prevention focus in their programs for families with young children. These programs include: Head Start [30]; Special Supplemental Nutrition Program for Women, Infants and Children (WIC) [31]; Supplemental Nutrition and Assistance Program–Education (SNAP–Ed) [32]; and Expanded Food and Nutrition Education Program (EFNEP) [33]. These four programs have a presence in all or most low-income communities in the U.S. Consequently, they have the potential to impact obesity prevalence among participants [34]. In response to the identified need for valid assessment tools targeting families enrolled in these federal programs, a pediatric obesity risk assessment tool, Healthy Kids, replete with visuals that replace and clarify text [35] making it suitable for families with limited written English literacy, was created using the results from two extensive literature reviews [36,37], and cognitive interviews with low-income English-speaking parents [38,39]. Healthy Kids was demonstrated to be reliable and valid using the objective measures, inflammatory biomarkers [40] and BMI [41], and subjective measures, 24-h dietary recalls and 36-h activity logs [41].

### Objectives

The current study is addressing this need for valid tools for low-income Spanish-speaking immigrant families in California. It is based on the above Healthy Kids research and includes visuals making it suitable for Hispanic families with limited English proficiency and a range of literacy skills in their main language, Spanish. In community settings, participant literacy drives the need for a tool with appropriate readability [42]. Evaluation and risk identification were two priorities for federal program participants while simultaneously having a low response burden for participants. Study objectives include selecting appropriate items from the pool of 45 items for a final parsimonious version, now called *Niños Sanos*, and establishing criterion validity of the final version of the *Niños Sanos* tool with low-income Spanish-speaking children and their families. Specifically, four hypotheses are tested: (1) *Niños Sanos* total score is associated with child body mass index (BMI) for-age percentiles and waist-to-height ratios with a lower *Niños Sanos* score predicting a higher BMI-for-age percentile; (2) *Niños Sanos* total score is associated with a child metabolic biomarker index with a lower *Niños Sanos* score indicating a less healthful, metabolic index score; (3) *Niños Sanos* total score is associated with a child lipid biomarker index with a lower *Niños Sanos* score indicating a less healthful, index score; and (4) *Niños Sanos* total score is associated with a child anti-inflammatory biomarker index with a higher *Niños Sanos* score indicating a more healthful, anti-inflammatory index score. Accordingly, we hypothesize that a lower *Niños Sanos* score, i.e., less healthful behaviors, is associated with children having a less healthful metabolic, lipid and inflammatory status and higher BMI compared to children with higher *Niños Sanos* scores.

## 2. Materials and Methods

### 2.1. Participants

Participants (*n* = 273 parent-child dyads) were recruited by direct solicitation and at information sessions at Head Start (*n* = 21) and WIC sites (*n* = 3) in 2 counties in northern California. The adults were parents or caregivers, ≥18 years, who declared Spanish as their preferred language, had at least one child aged 3–5 years and were low-income verified by participation in at least one federal assistance program for low-income residents.

### 2.2. Study Design, Timeline & Data Collection

Participants were recruited over the course of a year. For an individual parent/child dyad, data were collected in this 8-week cross-sectional study at 5 data collection points interspaced approximately 1–2 weeks apart as shown in Supplemental Table S1. At week 1, parents completed a demographic questionnaire as, well as the first child’s 36-h physical activity/screen time/bedtime logs and child’s 24-h dietary recalls [43]. The 36-h activity log and 24-h diet recall were administered again at weeks 5 and 7, for a total of 3 child 36-h activity logs and 3 child 24-h dietary recalls, the results of which will be reported elsewhere. The 45-item pediatric obesity risk assessment tool, *Niños Sanos* [44], was administered to parents at week 3 along with a food behavior checklist previously shown to be valid with Spanish-speaking immigrants [45,46,47]. Anthropometry and blood samples were collected from fasted children at the last time point around 8 weeks after the onset of week 1 of data collection. Each session lasted roughly one hour and data were collected in person and by phone interviews. All communications and documentation such as consent forms, assessment tools, questionnaires and logs were in Spanish and collected by trained research staff who spoke Spanish as a first language. The protocol, ID #693978-14, was approved by the Institutional Review Board of the University of California and participants provided a signed consent at enrollment and were given monetary stipends as compensation for their time and effort.

### 2.3. Biopsychosocial Framework

**Figure 1:**
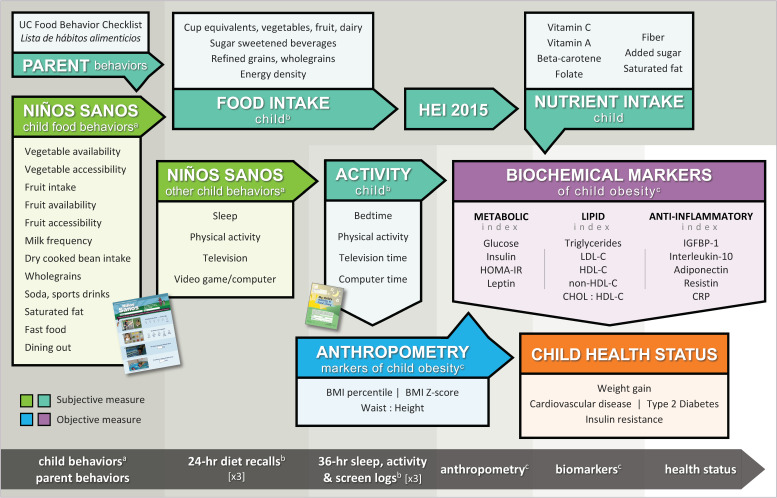
Biopsychosocial framework for validation of *Niños Sanos* targeting immigrant families with preschool-aged children. ^a^ Child dietary, physical activity, sleep and screen behaviors collected with original pool of 45-items for *Niños Sanos*. ^b^ Child diet estimated with 3 University of California Child Eating and Activity Diary and the National Cancer Institute’s Automated Self-Administered 24-h (ASA24) diet recalls. Child physical, screen, and sleep activities were estimated with 3 36-h activity logs using same Diary. ^c^ Child height, weight, waist, blood, blood pressure and body temperature were collected. Abbreviations: HEI: healthy eating index; ASA24: automated self-administered 24-h dietary assessment tool; HOMA-IR, homeostatic model assessment of insulin resistance; LDL-C, low density lipoprotein cholesterol; HDL-C, high density lipoprotein cholesterol; non-HDL-C, non-high density lipoprotein cholesterol; CHOL:HDL-C, cholesterol to high density lipoprotein cholesterol ratio; IGFBP-1, insulin-like growth factor binding protein-1; IL10, interleukin 10; CRP, C-reactive protein.

The framework used for the design of this validation research of *Niños Sanos* was based on systems theory [48,49]. This *Niños Sanos* framework considered the health of the child in the context of the family environment (Figure 1) and in that respect is similar to the Socio-Ecological Model (SEM) used to guide development of the tool’s content [50]. In hierarchical steps, this framework recognizes that the parent’s relevant behaviors create the environment for the 3–5 year old child and this, in turn, guides the child’s eating and activity behaviors [44]. In turn, the young child’s eating behaviors subsequently drive his/her food intake, measured by 3 National Cancer Institute’s automated self-administered 24-h (ASA24) diet recalls [51], resulting in intakes of macro and micronutrients. The parent’s control over the child’s physical, screen and sleep activities drives the child’s activity behaviors measured by 3 36-h activity logs [43]. Participant data obtained directly from the child employing objective measures are tinted blue and purple. Participant data collected from the parent about the child employing self-report/subjective measures are lime green and aqua. Thus, as specified by this *Niños Sanos* framework, intake of nutrients and physical activity/screen/sleep behaviors by the child can influence the child’s anthropometric and biochemical markers of childhood obesity, which then impacts the child’s health status. Figure 1 provides a graphic of the *Niños Sanos* framework.

### 2.4. Child Anthropometry

The child’s height, weight and waist were measured by trained observers using a portable stadiometer (Seca Road Rod, Seca Medical Measurement Systems & Scales, Hanover, MD, USA), a digital scale (Seca Carisma Model 810, Seca Medical Measurement Systems & Scales, Hanover, MD, USA) and body measuring tape (Creative Health Products, Ann Arbor, MI, USA). Children were measured in light clothing, no jackets or outerwear, without shoes. Weights were recorded to the nearest 0.1 kg and heights and waist circumferences were measured twice to the nearest 0.1 cm with the child fully erect, head in the Frankfort Plane and at the end of a deep inhalation. Waist measurement was taken at the narrowest portion of the torso, between the top of the child’s hip bone and lowest rib, bringing the tape measure around the body, level with the belly button, on children breathing normally. Waist-to-height ratio was calculated as 100 × waist (cm)/height (cm). Body mass index (BMI) (kg/m^2^) and waist-to-height (cm/m) ratio were calculated using the average of the measurements. BMI-for-age percentiles were derived using the Centers for Disease Control and Prevention (CDC)’s BMI percentile calculator for children [52]. BMI z-scores were calculated from the CDC pediatric growth charts using the CDC SAS codes [53]. CDC BMI-for-age percentile cut points were used to determine the weight status of each child (obesity, 95th percentile; overweight, 85th to less than the 95th percentile; normal weight, 5th percentile to less than the 85th percentile; and underweight, less than the 5th percentile).

### 2.5. Child Blood Biomarkers

For this study, an expert team selected biomarkers from the literature based on the association with child BMI. The biomarkers used for the current analyses are a subset and include: glucose, insulin, leptin, triglycerides (TG), high-density lipoprotein cholesterol (HDL-C), low-density lipoprotein cholesterol (LDL-C) and non-high-density lipoprotein cholesterol (non-HDL-C) [22,54]. Insulin-like growth factor binding protein-1 (IGFBP-1), interleukin-10 (IL-10), adiponectin, C-reactive protein (CRP) and resistin were also included [18,55,56,57]. Sources, targets and biological actions of these biomarkers as well as their relationship to children’s BMI are summarized in Table 1.

### 2.6. Calculated Biomarkers

LDL-C was calculated using the Friedewald equation as total cholesterol (mg/dL)—HDL-C (mg/dL)—TG (mg/dL)/5. Non-HDL-C is the total cholesterol minus HDL-C. HOMA-IR (homeostatic model assessment for insulin resistance) [58] was calculated as fasting insulin (μIU/mL) × fasting glucose (mmol/mL)/22.5. Cholesterol to HDL-C, triglycerides to HDL-C and leptin to adiponectin ratios were generated.

### 2.7. Blood Collection, Storage, Analysis

About 30 mL of blood was collected from fasted children into 3 vials via venipuncture by a certified pediatric phlebotomist from the University of California Davis Clinical Trial Center. Body temperature was taken to rule out potential illnesses as confounders. Vacutainers containing serum were left at room temperature 30 min to allow clotting and stored on ice for transport along with iced plasma tubes to the laboratory at the USDA Western Human Nutrition Research Center on the University of California at Davis campus, where the respective serum and plasma samples were recovered by centrifugation (15 min at 1500× *g* at 4 °C). Samples were aliquoted and stored at −80 °C for later analyses. Plasma glucose and lipids were measured by standardized enzymatic assays at the University of California Davis Medical Center. Serum insulin, leptin and adiponectin and plasma IGFBP-1 and IL-10 were measured by electro-chemiluminescent immunoassay using standard and custom single and multiplex panels (Meso Scale Discovery, MSD, Rockville, MD, USA). Biomarkers were analyzed in triplicates (IGFBP-1 in duplicates) with the selection of the best 2 of 3, if necessary, to reduce the coefficient of variability (CV) to less than 5% for all parameters (IGFBP-1, less than 10% CV). The multiplex assays required 7 plates for the analysis of the full data set. An internal control was added to each plate to adjust for plate-to-plate variability. There were no outliers, defined as values smaller or greater than 1.5 times the interquartile range above the upper quartile or below the lower quartile, in our data set.

### 2.8. Biomarker Indices

The biomarkers raw data were standardized using z-scores which enabled us to combine multiple biomarkers of different units into metabolic, lipid and inflammation indices. From a biological standpoint, the use of a cumulative index provides greater accuracy assessment of health status while decreased variability may increase the likelihood of a stronger correlation between variables than a single biomarker [59]. The metabolic index was the sum of z-scores of glucose, insulin, leptin, leptin to adiponectin ratio, triglycerides-to-HDL-C ratio and HOMA-IR. The lipid index was calculated as the sum of HDL-C (HDL-C z-scores multiplied by −1 to correct for its inverse association with the index), LDL-C, non-HDL-C cholesterol, cholesterol to HDL-C ratio and triglycerides. The anti-inflammatory index was the sum of the z-scores of adiponectin, IGFBP-1, IL-10, CRP and resistin. CRP and resistin z-scores were multiplied by −1 for inclusion in the anti-inflammatory index. The biomarker indices are summarized in Table 1.

### 2.9. Niños Sanos Cultural Adaptation and Face Validation

To ensure that *Niños Sanos* was acceptable for use by Spanish-speaking parents, Healthy Kids [60] was adapted following an iterative process with three components—forward translation, equivalence verification and cognitive interviews. The tool was forward translated by 2 bilingual and bicultural Spanish speakers and the photos were substituted with culturally appropriate images of families, activities and food items. Three content experts (i.e., child nutrition, developmental psychology) verified the translated tool to ensure it remained conceptually equivalent to Healthy Kids. The expert-verified tool was presented to Spanish-speaking parents who participated in one-on-one cognitive interviews. Twenty-three parents were recruited from 4 local Head Start preschool programs in a metropolitan area in Northern California and received $10 as compensation. After each round of cognitive interviews, changes to the tool were proposed and reviewed by content experts for equivalence verification. A new version that reconciled feedback from cognitive interviews and input from experts was created and used in a subsequent round of cognitive interviews with a new set of parents. This process was iterated four times until all items in *Niños Sanos* were deemed acceptable by both parents and content experts, at which point face validity was established.

### 2.10. Item Reduction for Niños Sanos

The 45 items on *Ninos Sanos* represented a pool of behaviors for 10 constructs compiled from 3 extensive literature reviews [36,37]. There were multiple items for each construct. To create this parsimonious tool appropriate for a low-income Spanish-speaking population in a community setting, item reduction was employed to produce a data driven, lower respondent burden version of *Niños Sanos* with 16–20 relevant items. Each of the items in the 45-item pool was coded on a 5-point scale, the highest score indicative of the healthier behavior, and discretized into categories. Boxplots were generated for each of the *Niños Sanos* 45 items and against the child anthropometric measures, i.e., BMI-for-age percentile, BMI z-scores and waist to height ratios. Items were deemed relevant if the boxplots showed a positive association with one or more of these anthropometric markers, i.e., when a higher score for a given item was associated with a lower BMI percentile-for-age, BMI z-score, or waist-to-height ratio. For example, the 45-item pool contained 9 fruit and vegetable items representing different fruit and vegetable behaviors published in the literature [37]; the 18-item tool contained 4 items for this construct.

### 2.11. Sample and Attrition

Families (*n* = 273) were enrolled in the study with 206 parent/child dyads completing the 5 data collection time points (to week 8), as shown in Supplemental Figure S1. The retention rate was 78%. At a significance level of *p* = 0.05, tests for attrition revealed that participants who remained in the study (*n* = 206) were not different for most demographic variables tested with two exceptions. Parents remaining in the study were more likely to report being unemployed compared to parents who dropped from the study (*n* = 67). In addition, parents in the study were slightly older than parents leaving the study (+2 years). Additional tests showed no difference to *Niños Sanos* scores between parents remaining in the study (*n* = 241) and those (*n* = 32) that dropped after week 1 (*p* = 0.63) (Supplemental Figure S1). From the final data set (*n* = 206), 202 parents completed all items on the *Niños Sanos* tool. Anthropometry was successfully measured on 200 children with some missing data due to fussy children or imprecise measures. Although 190 children donated a blood sample, the number of observations for biomarkers ranged between 159 to 163; missing data (*n* = 37–45) were due to insufficient amount of blood collected, parent refusal or child fussiness during blood draws and insufficient plasma and serum recovered during processing.

### 2.12. Statistics

Descriptive statistics were calculated as means with standard deviations and medians with interquartile range for continuous variables, or as frequencies and percent for categorical variables. Pearson’s Chi-squared test for association and t-tests were employed to compare demographic data between dropout and final samples. Logistic regression was used in a multivariate model to assess the effects of several covariates on dropouts. Sample size estimates for the main hypothesis of this study, a relationship of BMI-for-age percentiles with *Niños Sanos* high and low scores, were based on data from our previous work with English speaking families and Healthy Kids [41]. With at least 200 children, i.e., 100 in each group, this study was powered at 80% to detect a partial R^2^ of 0.08 or larger due to *Niños Sanos* scores. Boxplots of BMI-for-age percentiles were created across each of the 45 *Niños Sanos* items to illustrate the direction of the findings to define low and high *Niños Sanos* behavior scores. Preliminary analyses between the biomarker index z- scores and *Niños Sanos* showed that the relationships between these variables and the *Niños Sanos* high and low scoring groups were non-linear. Monotone transformations were tested using log, squared and square root. No transformation of the anthropometric and biomarker variables could be identified that provided an acceptable fit for a regression model with these data for the 18-item *Niños Sanos* scale; consequently, non-parametric tests were employed. The relationship between child anthropometry variables, i.e., BMI-for-age percentile, BMI z-scores and waist-to-height ratios, and biomarkers, i.e., metabolic, lipid and inflammation indices, with the 2 *Niños Sanos* groups were analyzed using Kruskal Wallace test. The range of scores spanned a narrow margin (51.5 to 80.5), i.e., only 29 points of a possible 18 to 90 or 69 points; it served the analyses better to consider 2 rather than 3 groups. Significance level was set at *p* ≤ 0.05 for all analyses. Statistical analyses were performed using SAS (SAS, version 9.4, SAS Institute, Cary, NC, USA).

## 3. Results

### 3.1. Demographics

Self-reported answers to the demographic questionnaire confirmed, as anticipated, that our cohort was predominantly low-income with Spanish as their first language, as shown in Table 2. All caregivers were female (100%) and most were not employed (71%). The majority reported earning a monthly household income of less than $2000 (57%). Additionally, families enrolled in the study participated in at least one USDA assistance program, with WIC (83%), Head Start (80%), Medicaid (70%) and SNAP (40%) being reported most frequently. All parents (100%) self-reported their and their child race/ethnicity as Hispanic/Latino. Most parents (81%) were born in Mexico, the majority (79%) had been living in the US for more than 10 years and 86% reported speaking Spanish at home. Parents were 33.6 ± 6 years old female (99%) married (65%) with a majority reporting having completed some high school or receiving a high school diploma (79%). Only 20% of the parents had a normal BMI with 80.5% having a BMI greater than 25. All children (54% female) were born in the US and were on average 44.7 months old at enrollment.

### 3.2. Item Reduction for Niños Sanos

The 18 items were selected based on their inverse association with child anthropometric boxplots; they are presented in Table 3 organized by behavioral domains or constructs. Item responses, means and standard deviations are presented with a maximum of five points representing the healthiest score. The subsequent validation analyses were conducted using this 18-item final version of *Niños Sanos* for a possible score ranging from 18 to 90 points. Although the lowest potential score was 18, the minimum reported score was 51.5 because few participants selected response options 1 and 2. The maximum reported score was 80.5. Using answers from the new version of *Niños Sanos* with 18 items, responses were scored and results were discretized into two groups of low and high scores (51.5 to to 74.9 points; 75.0 to 80.5 points) to adjust for skewness of the data in subsequent analyses. The range of scores for the individual items was fairly narrow with most item scores falling into the response options 3 and 4.

**Figure 2:**
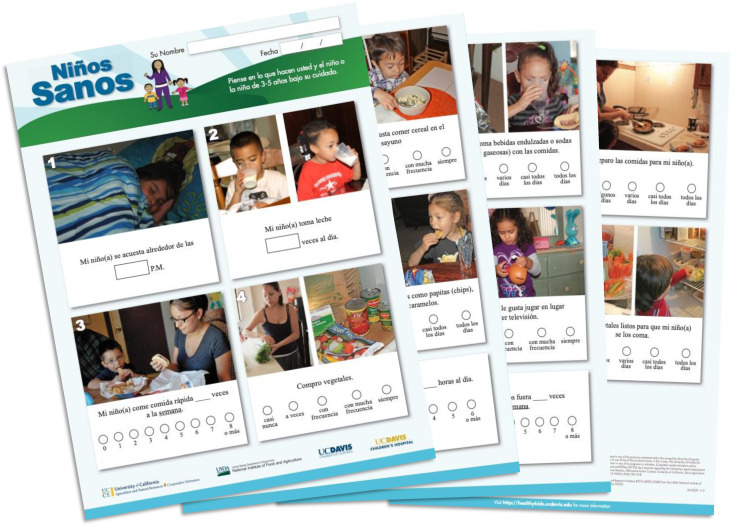
*Niños Sanos* Obesity Risk Assessment, final version with 18 items, tailored to Spanish speaking immigrant parents of 2–5 year old children. Printed in color on one 11 by 17-inch paper, folded in booklet format.

The constructs in the 18-item version included fruit availability and intake, vegetable availability and accessibility, eating dry cooked beans, milk intake, wholegrain intake, sugar sweetened beverages frequency, fast food frequency, trimming fat before eating, energy dense snacking, eating out, cooking from scratch, sedentary time, play vs. watching TV and sleep. The final version with 18 items is shown in Figure 2 [61].

### 3.3. Niños Sanos and Child Anthropometry

**Figure 3:**
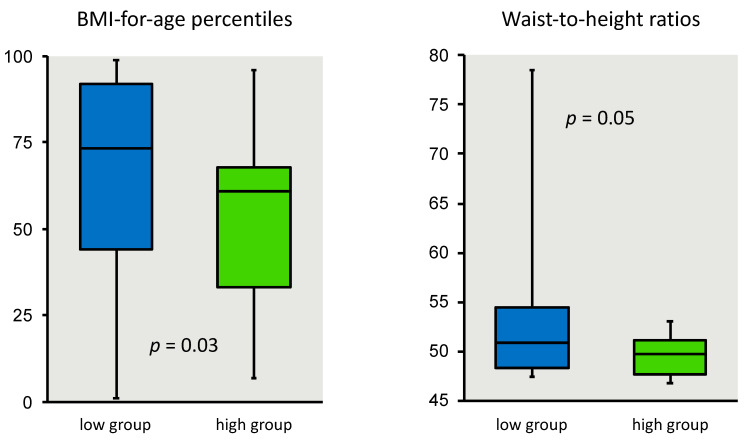
Child BMI-for-age percentile and waist-to-height ratios stratified by *Niños Sanos* groups of low and high scores. Answers on *Niños Sanos*, final version with 18 items, were scored and discretized into 2 groups of low and high scores (51.5 to 74.9 points; 75.0 to 80.5 points). Waist-to-height ratio was calculated as 100 × waist (cm)/height (cm).

A significant inverse relationship between the child’s 18-item *Niños Sanos* scores and BMI-for-age percentiles (*p* = 0.02) using Kruskal-Wallis is shown in Figure 3 and Table 4. The higher the child’s *Niños Sanos* score, i.e., healthier obesity prevention behaviors, the lower the child’s BMI-for-age percentile. Due to the skewness of the data, medians with interquartile range (IQR) instead of means with standard deviations are presented to summarize subgroup results (Table 4). Analysis using the child BMI z-scores, instead of BMI-for-age percentile, and *Niños Sanos* scores yielded the same inverse association with children in the high *Niños Sanos* scoring group having significantly lower BMI z-score than the children in the low scoring group (*p* = 0.03), also shown Table 4. In addition, high scores on the *Niños Sanos* were also predictive of lower waist-to-height ratios in children (*p* = 0.05) as shown in Figure 3 and Table 4. We repeated the analysis using analysis of variance; similar statistical results were found.

### 3.4. Child Biomarkers and Anthropometry

There was a significant positive association between the metabolic and lipid biomarker indices and the underweight, normal and overweight BMI categories. In contrast, the anti-inflammatory index was inversely associated with BMI categories (Table S1).

### 3.5. Niños Sanos and Child Biomarker Indices

**Figure 4:**
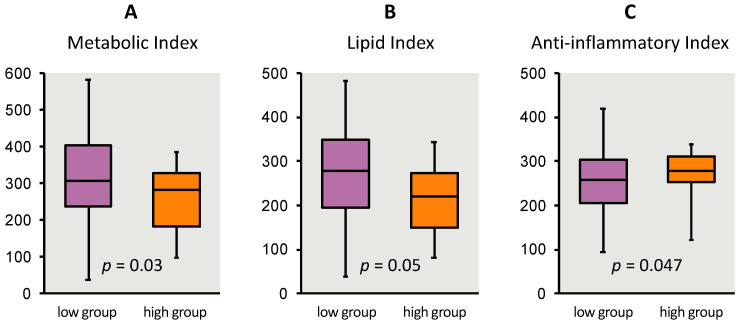
Child biomarker indices for metabolic (**A**), lipid (**B**) and anti-inflammatory (**C**) stratified by *Niños Sanos* groups of low and high scores. Answers on *Niños Sanos,* final version with 18 items, were scored and discretized into 2 groups of low and high scores (51.5 to 74.9 points; 75.0 to 80.5 points). Metabolic index (**A**) composed of glucose, insulin, leptin, leptin:adiponectin. Lipid index (**B**) composed of triglycerides, high density lipoprotein (HDL) cholesterol, low density lipoprotein cholesterol, cholesterol:HDL cholesterol. Anti-inflammatory index (**C**) composed of Interleukin 10, adiponectin and IGFBP-1 insulin growth factor binding protein, resistin and C-reactive protein.

As hypothesized, circulating child blood levels of biomarkers grouped into metabolic and lipid indices were significantly higher in children with low *Niños Sanos* scores, while high scores predicted lower metabolic (*p* = 0.03) and lipid indices (*p* = 0.05). In addition, a high *Niños Sanos* score was associated with a higher anti-inflammatory index reflecting a more healthful status (*p* = 0.047) (Figure 4, Table 4).

## 4. Discussion

This research paper describes the quantitative research to establish criterion validity of *Niños Sanos* using objective parameters collected from young children, 3–5 years old, of Spanish-speaking immigrant parents, including those with limited written Spanish literacy. The relationship was statistically significant between *Niños Sanos* scores and three anthropometric measures that included BMI-for-age percentile, BMI Z-scores and waist-to-height ratios and three blood biomarker indices of obesity that included a metabolic index, lipid index and anti-inflammatory index as shown in Table 4 and Figure 3. Children in the high scoring group for *Niños Sanos*, i.e., the healthier scores, had lower BMI-for-age percentile, BMI z-scores and waist-to-height ratios and significant healthful metabolic, lipid and inflammatory profiles. Conversely, children with lower *Niños Sanos* scores showed compromised health status. The results suggest that a tool like *Niños Sanos* is useful to identify children of Spanish-speaking immigrant families who are at risk of impaired weight and metabolic outcomes based on the environment and behaviors practiced at home. Combining these metabolic, lipid and inflammatory indices with the results for BMI-for-age percentiles, BMI z-scores and waist-to-height ratios creates a broad picture of the child’s health status. Moreover, *Niños Sanos* is related to all these variables indicating that criterion validation is established for the tool. Those children with higher scores have a better health profile both metabolically and anthropometrically than children in the lower scoring group.

### 4.1. Anthropometric and Blood Biomarkers in Children

Children and particularly children of Mexican descent may be at risk for metabolic complications and anthropometric parameters may not be as sensitive to the changes in visceral fat accumulation as circulating levels of adipokines and other metabolic parameters [9,62]. The inclusion of biomarkers may provide additional means to identify children with obesogenic family environments. Individually, circulating levels of biomarkers measured in our study compare reasonably to reference values published in the literature (Supplemental Table S1). As minimal biomarker research has been undertaken with young children, it was not clear to us at what age biomarkers of obesity could be used to validate an obesity prevention risk assessment tool. However, it would appear from our data that ages 3–5 years is not too young.

In our study, children with high BMI-for-age percentiles show an unfavorable blood lipid profile. Longitudinal studies have shown that children with high BMI have increased risk of cardiovascular disease in adulthood [63]. Studies with large cohorts of Hispanic children indicate that pre and post pubertal children with overweight or obesity have significantly higher levels of TG and LDL-C and lower circulating HDL-C than normal weight peers [64,65]. This is relevant for children of Mexican descent who because of genetic predisposition have been found to be at a greater risk for early onset of adverse health complications [62]. It is a concerning observation as dyslipidemia leading to atherosclerosis may begin in childhood for children with obesity [24].

Although we did not screen specifically for the prevalence of obesity in our child participants, it was higher at 19% than the US obesity rates for 2–5 year olds at 14%. Moreover, these overweight and obese children exhibited high HOMA-IR and leptin to adiponectin ratio, exceeding the published cut off levels for children that age. As observed in adults with obesity, our data as well as that of other researchers indicate that elevated insulin or insulin resistance is not uncommon in Hispanics children with obesity [54,66]. In pre-pubertal children with obesity, low levels of circulating IGFBP1 and adiponectin are strong predictors of insulin resistance and metabolic complications [54,67,68]. Both biomarkers were lower in children with overweight and obesity in our study, while leptin levels were elevated. These 2 conditions (high leptin and low adiponectin) appear to be important markers of body fat and of comorbidity risk in children [12,16,66]. Overall, our results support that these selected biomarkers were sensitive to obesogenic behaviors and likely reflect body visceral fat accumulation. Thus, these biomarkers were useful for the validation of *Niños Sanos.*

### 4.2. Advancing Obesity Risk Assessment

Advancing the science of obesity risk assessment requires integrated approaches that deploy multiple types of validation and determine alignment with objective measures [69]. Public health experts have called upon the behavioral sciences to consider a more integrated approach to better understand how obesogenic behaviors interact. Instead of assessing one behavior in isolation, it is recommended to measure multiple co-dependent obesogenic behaviors simultaneously [69]. Exploring the integration of obesity related behaviors like the time children spend active, sedentary and sleeping and the synergy on childhood obesity is regarded as a paradigm shift from current practice. The present study is an example of a validation research using objective standards, such as BMI and blood biomarkers, to determine the usefulness of a self-report tool that encompasses the assessment of multiple behaviors—various dietary components, physical activity, sleep and screen time—to identify children at risk of obesity and related diseases. In addition, validation studies have traditionally focused on validating obesity-related self-report behavioral tools using other subjective self-report measures, such as the 24 h diet recall or food frequency questionnaire [70]. Another paradigm shift has occurred over 20 years, emphasizing the need for objective assessments of obesogenic behaviors to validate short self-report behavioral tools, in addition to the more traditional self-report measures. This was accomplished through the introduction of biologic objective measures [70], such as blood values to assess chronic low-grade inflammation [40], serum carotenoids to validate fruit and vegetable intakes [42], body mass index and other anthropometric markers [71], videotaping family meals to measure parenting practices [72], digital photography to measure dietary intake [73] and actigraphy devices to measure physical activity, sedentary behavior and sleep. The emphasis in all these methods are objective measurements to support or replace the more traditional parent-report methods. In addition to objective measurements for validation, there is more interest in incorporating multiple approaches to get a more complete picture of the validation process [69]. Each type of validation provides somewhat different information and adds to this total validation picture. Likewise, each type of reliability assessment provides different information. Reliability results for *Niños Sanos* will be reported elsewhere.

### 4.3. Comparison to Other Validation Studies for This Audience

For comparison, our previous [36,37] and more recent reviews of the literature found no validation studies supporting obesity prevention tools for low-income, Spanish-speaking families with young children. However, there were two similar tools reported in the literature targeting English-speaking families with young children. The 21-item Family Nutrition and Physical Activity (FNPA) screening tool for English-speaking families with children 6–12 years was designed for school and medical clinic settings and was extensively validated with objective measures including anthropometric and blood biomarkers [74,75] and translated into Spanish. A second tool was cognitively tested with Spanish speakers to establish face validity for use with EFNEP evaluation, and by comparing it to other self-report behavioral assessments for diet, lifestyle and parenting [76]. *Niños Sanos* is the only pediatric obesity risk assessment tool designed specifically for Spanish-speaking parents and rigorously validated with objective measures, including anthropometry and blood biomarkers. Given the high prevalence of overweight and obesity among the Hispanic population in the U.S., a tool such as this was long overdue.

### 4.4. Beyond Translation

The original 45-item *Niños Sanos* was tested to ensure conceptual equivalence with the original 45-item English version Healthy Kids. In both tools, selection of items for their respective final versions was driven by the relationship with anthropometric markers of childhood obesity. Nonetheless, the set of items identified to be criterion-relevant differed substantially between both tools, with only eight overlapping items. The vegetable domain, for instance, had nine items in both 45-item versions. Five of the nine items performed well with BMI for English speakers, thus were included in the 19-item risk assessment version of the tool. In contrast, only two of the nine items were associated with anthropometric measures among Spanish speakers, and only one of them performed well with both audiences—the vegetable availability item. Vegetable accessibility was found relevant for Spanish speakers, while vegetable modelling at mealtime, vegetable snacks and vegetable variety items were important for the English but not Spanish speakers. These findings are clear evidence of the need to apply the same level of exhaustiveness and rigor to the validation of tools intended for culturally and linguistically specific audiences. While useful to establishing feasibility of reach, forward and back translations are always insufficient to ensure the content relevance and accuracy of a tool, including its usefulness at identifying those at risk of malnutrition and related conditions [77,78]. Practitioners for community nutrition education programs with mixed audiences may find using two different tools for program evaluation impractical. However, matters of convenience should be considered against the fact that data-driven valid tools are essential for accurate and meaningful evaluation.

### 4.5. Comparison to Other Biomarker Literature

Using the Framework (Figure 1), these data show that the *Niños Sanos* tool is valid to use for risk assessment in Hispanic families with 2–5 year-old children. To support the strength and direction of our findings, biomarkers and *Niños Sanos* tool, we examined other biomarker studies, some of which were interventions. Children with overweight or obesity have low circulating interleukin-10 concentrations, also found by Chang et al. [18]. In pre-pubertal children with obesity, low circulating IGFBP-1 is a strong predictor of insulin resistance and metabolic complications [67].

### 4.6. Using Niños Sanos

The *Niños Sanos* behavioral assessment for Spanish-speaking immigrant families has culturally appropriate color photographs demonstrating each behavior to minimize cognitive load. This assessment format is optimal for audiences with limited literacy and for those immigrating from Spanish-speaking countries and whose English proficiency is limited [35]. *Niños Sanos* takes substantially less time for participants to complete than 24 h diet recalls and does not need a professional for data collection and interpretation. Tailored goals can be generated from the *Niños Sanos* results available at http://healthykids.ucdavis.edu/ to assist parents with goal setting during an intervention or counseling session for families with children most at risk for metabolic disturbances and excess weight gain [79,80]. This tool could serve other functions as well. First, it could be used in research studies targeting low-income Spanish-speaking audiences. *Niños Sanos* could also serve as an evaluation to assess program or intervention impact. Lastly, medical providers could benefit from a risk assessment tool during well-child visits as recommended by the American Academy of Pediatrics [4,5,6,81,82].

### 4.7. Limitations and Strengths

This study had multiple strengths including use of objective robust analytical approaches to establish validity of *Niños Sanos* (Figure 1) not subject to self-report bias, i.e., direct measurement of height, weight and waist by physical examination and collection of blood for measurement of metabolic, lipid and anti-inflammatory biomarker values; these markers provide confidence in the estimates and outcomes generated by this research [70]. Another strength is the sample of low-income Hispanic children at high risk for obesity.

At the same time, a number of limitations should be noted. The biomarker reference values available from the literature and cited in the Supplemental Table S1, were based on data available for non-Hispanic white children, not other racial/ethnic groups. Although the external validity of these findings to other Spanish-speaking immigrant audiences is unknown, the methods applied to the development of the tool are consistent with theories of visual information processing [83] and cross-cultural adaptation principles [84], with efforts to reduce client cognitive load for those federal program participants with a broad range of literacy skills [85]. As parents volunteered to participate and were not randomly drawn from the larger low-income Spanish-speaking WIC and Head Start populations, the variability of their responses may be smaller compared to that of the general limited-resource target population. Consequently, selection bias must be considered as a threat to external validity [86]. Only a small portion of the children in the study scored high in the *Niños Sanos* tool (i.e., *n* = 20). Despite the impact on the statistical power this could have posed, the differences between the two groups were statistically significant for BMI and z-scores indicating that the differences were real. Similarly, for the biomarker analyses, even as the sample size was constrained by the number of participants with a blood sample sufficient in quantity for each type of biomarker analysis, we were able to detect statistical significance on the metabolic, lipid and anti-inflammatory indices. The boxplot for each of the three biomarker indices (Figure 3) indicates children’s values moving in predicted directions.

## 5. Conclusions and Implications

To our knowledge, this is the first study establishing criterion validity for a pediatric obesity assessment tool targeting Spanish-speaking immigrant families with young children. *Niños Sanos* has potential as a rapid and easy-to-administer parent assessment of the young child’s risk of overweight or obesity in a community education or medical clinic setting, as an evaluation assessment of a corresponding education intervention or in a research setting. The validation results show statistically significant relationships between *Niños Sanos* and BMI-for-age percentiles, BMI z-scores, waist-to-height ratio, and with metabolic, lipid and inflammatory profiles.

Implications of this *Niños Sanos* validation study include contributing to the body of literature on the use of objective biometric measures for robust validation of self-report behavioral measures and to expand their value to risk detection. Researchers can use the study to encourage the use of blood biomarkers with young children for future validation research. The study also provides evidence that a tool intended to be used with audiences culturally different from which it was originally created requires thorough validation with objective indicators to ensure behaviors are accurately measured for plausible risk detection. Lastly, to further address the complexity of childhood obesity risk, a parenting practices assessment, *My Child at Mealtime* and its culturally adapted version, *Mi Niño a la Hora de Comer* validated with Spanish-speaking parents, developed by the same research group, is intended for use in tandem with *Niños Sanos* [71,72,87].

## Figures and Tables

**Table 1:** Characteristics and evidence for the selection of the biomarkers included in the metabolic, lipid and anti-inflammation indices.

	Sources	Targets	Biological Actions	Relationship to BMI in Children
**Metabolic index**				
**Glucose**	Diet, Liver, Muscle	Cells	Energy	Positive [22]
**Insulin**	Pancreas	Muscle, AT	Glycemia homeostasis, lipolysis inhibition	Positive [22,54]
**HOMA-IR**	n/a	n/a	Insulin sensitivity index	Positive [22,54]
**Leptin**	AT	Brain (hypothalamus), Muscle, AT, liver	Regulation of food intake, satiety and energy expenditure	Positive [22]
**Leptin: Adiponectin**	n/a	n/a	Adipose tissue dysfunction index	Positive [55]
**TG: HDL-C**	n/a	n/a	Insulin resistance index	Positive [56]
**Lipid index**				
**Triglycerides**	(Diet), Liver, AT	AT	Energy	Positive [22,54]
**LDL-C**	Plasma	Cells, Liver	Cholesterol transport (influx)	Positive [22]
**HDL-C**	Plasma	Liver	Cholesterol transport (effux)	Negative [22,54]
**Non-HDL-C**	Gut, Liver	Cells, Liver	Cholesterol transport (influx)	Positive [22]
**CHOL:HDL-C**	n/a	n/a	Pro-atherogenic index	Positive [22]
**Anti-inflammatory index**				
**IGFBP-1**	Liver	Muscle, AT	Insulin sensitivity, anti-inflammatory	Negative [57]
**Interleukin-10**	AT, Spleen	Liver	Anti-inflammatory	Negative [18]
**Adiponectin**	AT	Pancreas, Muscle, Liver, AT	Anti-inflammatory, insulin sensitivity	Negative [22,54]
**CRP**	Liver	Muscle, AT	Pro-inflammatory, insulin resistance	Positive [22]
**Resistin**	AT	AT	Insulin resistance, food intake	Positive [14]

Abbreviations: AT, adipose tissue; HOMA-IR, homeostatic model assessment of insulin resistance; LDL-C, low density lipoprotein cholesterol; HDL-C, high density lipoprotein cholesterol; TG: HDL-C, triglyceride: high density lipoprotein cholesterol ratio; non-HDL-C, non-high density lipoprotein cholesterol; CHOL:HDL-C, cholesterol to high density lipoprotein cholesterol ratio; IGFBP-1, insulin-like growth factor binding protein-1; IL10, interleukin 10; CRP, C-reactive protein.

**Table 2:** Descriptive statistics of *Niños Sanos* study participants by parent, child, family and acculturation variables (*n* = 206 parent/child dyads).

**Demographic Characteristics**					
**Parent/Guardian ^a,b^**		**Child ^a,b^**		**Family/Household ^a,b^**	
**Gender (Female)**	206 (100)	**Gender (Female)**	113 (54.3)	**Household Income** (monthly)	
**Age** (years)	33.6 ± 6.0	**Age** (months)	51.8 ± 8.0	<$2000	118 (56.7)
**Marital status**				$3000–3500	13 (6.3)
Married	136 (65.4)			$3500–4000	5 (2.4)
**BMI**		**BMI-for-age-percentile**		>$4000	5 (2.4)
Normal <25	40 (19.5)	Underweight <5th %ile	8 (4.0)		
Overweight (25–30)	75 (36.6)	Normal weight <85th %ile	130 (65.0)	**Assistance programs** ^c^	
Obesity (30–40)	77 (37.6)	Overweight > 85th %ile	24 (12.0)	Head Start	166 (79.8)
Severe obesity >40	13 (6.3)	Obesity >95th %ile	38 (19.0)	WIC	172 (82.7)
**Education**				SNAP	84 (40.4)
College degree	13 (6.4)			TANF	18 (8.6)
Some college	30 (14.7)			NSLP	62 (29.8)
High school diploma	59 (28.9)			Head Start	166 (79.8)
**Employment**				WIC	172 (82.7)
Unemployed	148 (71.1)				
Seasonal	35 (16.8)				
Full time	25 (12.0)				
**Acculturation Components** ^a,b^					
**Parent/guardian** ^a,b^		**Child** ^a,b^		**Family/household** ^a,b^	
**Living in U.S. (years)**				**Language spoken at home**	
<3	7(3.4)			English	13 (6.3)
3–9	33 (16.2)			Spanish	179 (86.1)
10–20	116 (56.9)			English or	16 (7.7)
>20	48 (23.5)			Spanish	
**Country of Birth**		**Country of Birth**			
U.S.	0 (0)	U.S.	198 (95.2)		
Mexico	168 (80.8)	Mexico	7 (3.4)		
Other	40 (19.2)	Other	3 (1.4)		
**Ethnicity**		**Ethnicity**			
Hispanic/Latino	100	Hispanic/Latino	98.6		

^a^ Categorical data are reported as number of participants (% of sample). ^b^ Continuous data are reported as means (± standard deviations). ^c^ The major requirement for program participation is income using a formula factoring in household size and income. Note: all parent variables are shaded blue. All child variables are shaded purple. All family variables are shaded orange. Abbreviations: TANF, Temporary Assistance for Needy Families; NSLP, National School Lunch Program; WIC, Supplemental Nutrition Assistance for Women, Infants and Children; SNAP, Supplemental Nutrition Assistance Program.

**Table 3:** Behavioral domain and construct, item text, item visual content and means ± SD for 18 items selected for final version of *Niños Sanos* assessment tool.

Behavioral Domain & Construct	Item Text	Item Visual	Response ^a^
**Vegetables**			
Vegetable availability	*Compro vegetales*. I buy vegetables.	Left: woman getting ready to buy broccoli with her daughter. Right: bag of frozen mixed vegetables, box of frozen green beans, can of diced tomatoes, can of corn and can of mixed vegetables.	4.2 ± 1.0
Vegetable accessibility	*Tengo vegetales listos para que mi niño (a) se los coma*. I keep vegetables ready for my child to eat.	Left: refrigerator shelf with bowl of washed cherry tomatoes, carrot and celery sticks in a glass, carrot sticks/celery sticks/cherry tomato in snack bag. Right: child reaching for vegetable snack on refrigerator shelf.	3.2 ± 1.3
**Fruit**			
Fruit intake	*Yo como frutas __ veces al dia*. I eat fruit ____times a day.	Left: mother biting into an apple. Right: another mother eating a banana.	3.3 ± 0.9
Fruit availability	*Compro frutas*. I buy fruit.	Left: mother biting into an apple. Right: another mother eating a banana.	4.4 ± 0.8
**Beans**			
Dry cooked bean intake	*Mi niño (a) come frijoles ___ veces por semana*. My child eats beans ____times a week.	Left: dry beans as purchased, cooked dry beans in cans. Right: mother and daughter who is serving prepared beans.	2.4 ± 1.0
**Dairy**			
Milk frequency	*Mi niño(a) toma leche __ veces al dia*. My child drinks milk ___ times a day.	Left: parent pouring milk on cereal. Center: Boy drinking milk in glass with snack/meal. Right: boy drinking chocolate milk via a straw.	3.3 ± 0.8
Milk frequency	*Yo tomo leche __ veces al dia*. I drink milk ___ times a day.	Left: mother drinking milk from a glass. Right: another mother drinking milk from a glass.	2.3 ± 0.8
**Whole Grains**			
Milk with cereal	*A mi nino(a) le gusta comer cereal en el desayuno*. My child enjoys cereal for breakfast.	Child with cereal and empty glass of milk.	3.0 ± 1.2
**Sugar Sweetened Beverages**			
Soda frequency	*Mi niño(a) toma sodas __ veces al día*. My child drinks soda ____times a day.	Left: girl drinking Mexican soda from a bottle. Right: selection of carbonated beverages in cans, bottles and paper cup from Mexico and U.S.	4.7 ± 0.4
Sports drinks, punch frequency	*Mi niño(a) toma bebidas deportivas o endulzadas __ veces al día*. My child drinks sport drinks or sugared drinks ___ times a day.	Left: boy drinking Kool-Aid© from disposible pouch. Right: SunnyD©, Hawaiian Punch©, Propel Fitness Water©, Gatorade©, Kool-Aid©.	4.5 ± 0.6
**Fat/Saturated Fat**			
Energy density	*Mi niño(a) come comida rápida __ veces a la semana*. My child eats fast food _____times a week.	Child eating hamburger from fast food outlet. Also shown are French fries and soda in paper cup with straw from a Happy Meal box.	4.4 ± 0.4
Fat, energy density, saturated fat	*Le quito la grasa a la carne antes de comerla*. I trim fat before eating.	Parent’s hand with knife trimming fat from raw meat on cutting board. Parent’s hand with fork and knife trimming fat from cooked meat as served on dinner plate.	4.3 ± 1.1
**Snack Foods**			
Energy dense foods for snack	*Mi nino(a) come snacks como papitas (chips), galletas y dulces*. My child eats snack foods like cookies, chips and candy.	Left: boy eating cookie. Center: girl eating Mexican pastry Right: girl eating chips.	4.0 ± 0.7
**Eating Out**			
Energy density	*Nosotros comemos fuera __ veces a la semana*. We eat out ____times a week.	Two parents with young child sitting at table in restaurant eating burritos and soda.	4.1 ± 0.8
**Cooking at Home**			
Energy density	*Preparo las comidas para mi niño(a)*. I cook my child’s dinner from scratch.	Parent at stove cooking meat in skillet with young child watching.	4.6 ± 0.7
**Screen Time**			
Television	*Mi niño(a) mira la televisión __ horas al dia*. My child watches TV ___ hours a day.	Girl watching TV in living room/front room.	3.7 ± 0.6
**Physical Activity**			
Play, sedentary time	*A mi niño(a) le gusta jugar en lugar de ver televisión*. My child likes playing instead of watching TV.	Girl playing with toy in her bedroom.	3.4 ± 1.1
**Sleep**			
Bedtime	*Mi niño(a) se acuesta alrededor de las __ PM*. My child goes to bed around ___ p.m.	Young girl asleep in her bed in child’s dark bedroom.	3.0 ± 0.8

^a^ Each item has a minimum of 1 and maximum of 5 points; responses are as means ± standard deviations. The colored rows contain the behavioral domain while the text immediately under with no color is the construct.

**Table 4:** Child anthropometric and blood biomarkers by index (metabolic, lipid and anti-inflammatory) stratified by low and high scores for *Niños Sanos* participants.

	*Niños Sanos*										*p*-Value ^a^
	Low Scoring Children					High Scoring Children					
	*n*	Median	IQR	Q1	Q3	*n*	Median	IQR	Q1	Q3	
**Anthropometric**											
BMI percentiles-for-age	176	73.50	48.00	44.00	92.00	20	61.00	35.00	33.00	68.00	**0.024**
BMI Z-scores	176	0.65	1.51	−0.12	1.39	20	0.29	1.01	−0.45	0.56	**0.028**
Waist-to-height ratios	176	50.85	6.08	48.35	54.43	20	49.65	3.45	47.71	51.16	**0.053**
**Metabolic**											
Metabolic index	141	307.07	166.86	237.11	403.96	18	281.08	145.27	180.90	326.17	**0.028**
Lipid index	149	263.96	152.66	195.27	347.93	18	219.23	121.89	150.30	272.19	**0.050**
Anti-inflammatory index	140	258.39	96.25	206.05	302.31	18	277.20	55.83	253.99	309.82	**0.047**

*^a^. Kruskal Wallis.* Bold text in left column show variable categories. Others without bold are variable sub-categories. *p*-values are shown in right-hand column in bold with light mauve shading.

## Supplementary Materials

The following are available online at https://www.mdpi.com/2072-6643/12/11/3582/s1, Figure S1: Flow diagram of initial recruits and subsequent dropouts of parent/child dyads at each stage of data collection for the *Niños Sanos* validation study, Table S1: Biomarkers (metabolic, lipid and anti-inflammation index) and range values, stratified by children BMI-for-age percentiles (ranks) for *Niños Sanos* study participants.
