# Supplementary material for: Obesity Risk Assessment Tool for Low-Income Spanish Speaking Immigrant Parents with Young Children: Validity with BMI and Biomarkers of Obesity
Source: Nutrients. 2020 Nov 22;12(11):3582. doi: 10.3390/nu12113582 (PMC7700341; doi:10.3390/nu12113582)
Supplement: Supplementary file 1 [file nutrients-12-03582-s001.pdf]

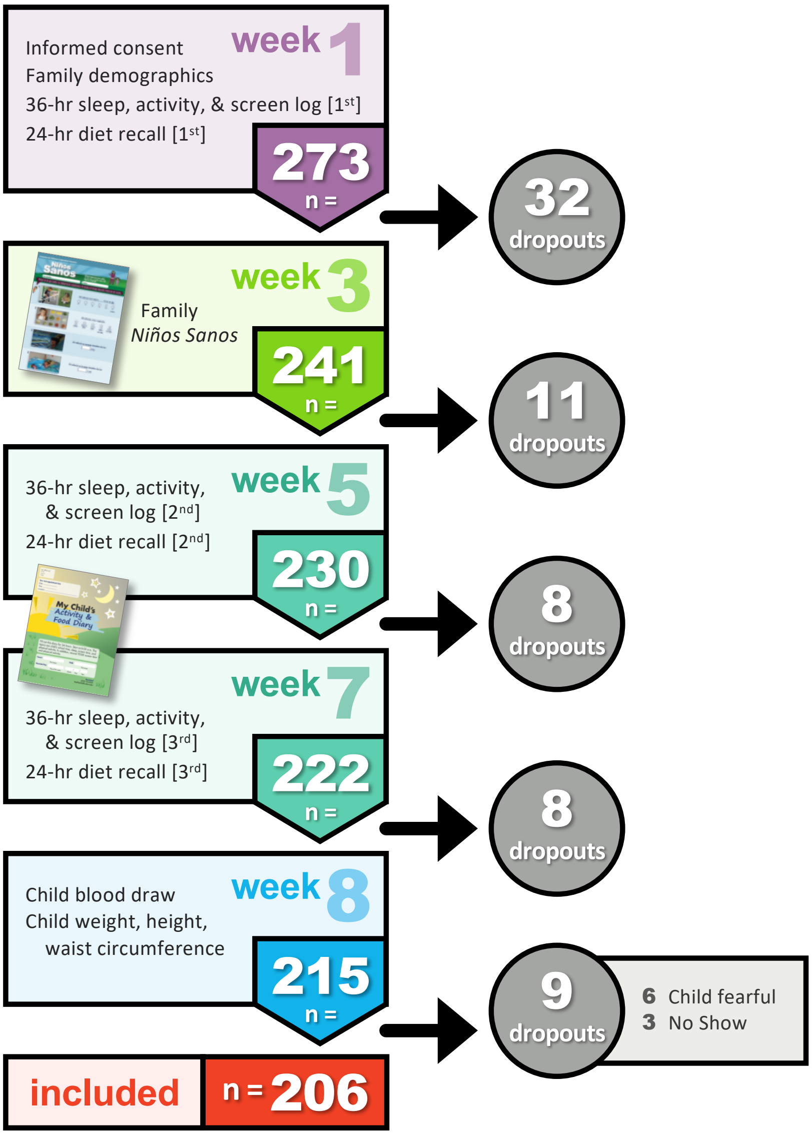

Figure S1. Flow diagram of initial recruits and subsequent dropouts of parent/child dyads at each stage of data collection for the *Niños Sanos* validation study.

Table S1. Biomarkers (metabolic, lipid and anti-inflammation index) and range values, stratified by children BMI-for-age-percentiles (ranks) for *Niños Sanos* study participants.

BMI-for-age percentiles	Underweight			Normal weight			Ow/Ob			*p*-value	Cut off values (age) Reference values (age)
	n	Mean	SD	n	Mean	SD	n	Mean	SD		
**Metabolic Index**											
Glucose	7	71.57	9.36	111	73.86	6.99	50	75.30	6.58	NS	65-100 mg/dl (5-9)[1]
Insulin	6	2.78	0.76	111	3.45	1.52	50	6.08	4.22	0.0001	<13.7 uUI/L (5-9)[1] 13-24 uUI/L (3-6)[2]
Leptin	6	0.76	0.17	109	1.56	1.13	50	4.95	5.07	0.0001	0.6-16.8 ng/ml (5-9)[1] 10.8-12.7 ng/ml (3-6)[3]
HOMA-IR	6	0.30	0.15	108	0.63	0.30	48	1.14	0.78	0.0001	1.0 (0.5-1.4)[2] 0.48-0.70 (3-6)[2]
Leptin: Adiponectin	6	3.47	1.32	109	8.09	6.35	50	30.39	36.86	0.0001	n/a 0.5-2.6 (6-18)[4]
TG:HDL-C	7	0.92	0.39	111	1.29	0.73	50	1.59	0.56	0.0037	n/a 0.33 to 3.21 (10-16) [5]
**Lipid Index**											
HDL-C	7	55.29	14.20	111	51.82	10.85	50	51.08	7.39	NS	> 45 mg/dl (2-17)[6] 44-51 mg/dl (3-6)[7]
LDL-C	7	79.14	10.24	111	90.09	22.44	50	94.12	19.65	NS	< 110 mg/dl (2-17)[6] 93-95 mg/dl (3-6)[7]
CHOL: HDL-C	7	2.70	0.45	111	3.08	0.62	50	3.17	0.44	0.0300	<3.8 mg/dl (2-17)[6]
Non-HDL-C	7	88.86	11.25	111	103.50	23.39	50	108.94	20.77	0.0260	n/a n/a
Triglycerides	7	48.14	16.94	111	62.51	28.41	50	74.01	24.75	0.0005	75 mg/dl (2-9)[6] 45 mg/dl (3-6)[7]
**Anti-inflammatory Index**											
Adiponectin	6	24.86	9.69	110	22.01	8.81	50	21.91	10.97	NS	4-37 ug/ml (2-17)[6] 11.1-13.4 ug/ml (3-6)[3]
IGFBP-1	6	317.35	131.02	111	244.88	132.28	50	149.29	83.39	0.0001	30-100 ng/ml [6] 50-102 ng/ml (5-15)[8]
Interleukin-10	6	0.68	0.90	111	1.11	2.62	49	0.64	0.82	NS	<9.2 pg/ml (2-17)[6]
CRP	6	0.84	1.88	110	1.53	5.20	47	2.70	6.22	0.0017	<10 mg/L 0.5 mg/L(0.3-10.7)[9]
Resistin	6	1.38	0.34	110	1.73	0.73	50	1.63	0.58	NS	n/a 3.25-26.4 ng/ml [10]

Abbreviations: Ow/Ob, Overweight/Obesity; HOMA-IR, Homeostatic Model Assessment of Insulin Resistance; LDL-C, Low density lipoprotein cholesterol; HDL-C, high density lipoprotein cholesterol; TG:HDL-C, triglyceride:high density lipoprotein cholesterol ratio; non-HDL-C, non-high density lipoprotein cholesterol; CHOL:HDL-C, cholesterol:high density lipoprotein cholesterol ratio; IGFBP-1, Insulin-like growth factor binding protein-1; CRP, C-reactive protein.

References

1. Styne, D.M. *Pediatric Endocrinology: A Clinical Handbook*; Springer International Publishing: New York, NY, USA, 2016; ISBN 978-3-319-18370-1.

2. Peplies, J.; Jiménez-Pavón, D.; Savva, S.C.; Buck, C.; Günther, K.; Fraterman, A.; Russo, P.; Iacoviello, L.; Veidebaum, T.; Tornaritis, M.; et al. Percentiles of fasting serum insulin, glucose, HbA1c and HOMA-IR in pre-pubertal normal weight European children from the IDEFICS cohort. *Int. J. Obes.* **2014**, *38*, S39–S47, doi:10.1038/ijo.2014.134.
3. Erhardt, E.; Foraita, R.; Pigeot, I.; Barba, G.; Veidebaum, T.; Tornaritis, M.; Michels, N.; Eiben, G.; Ahrens, W.; Moreno, L.A.; et al. Reference values for leptin and adiponectin in children below the age of 10 based on the IDEFICS cohort. *Int. J. Obes.* **2014**, 38 (Suppl. 2), S32–S38, doi:10.1038/ijo.2014.133.
4. Frithioff-Bøjsøe, C.; Lund, M.A.V.; Lausten-Thomsen, U.; Hedley, P.L.; Pedersen, O.; Christiansen, M.; Baker, J.L.; Hansen, T.; Holm, J.-C. Leptin, adiponectin, and their ratio as markers of insulin resistance and cardiometabolic risk in childhood obesity. *Pediatr. Diabetes* **2020**, *21*, 194–202, doi:10.1111/pedi.12964.
5. Nur Zati Iwani, A.K.; Jalaludin, M.Y.; Wan Mohd Zin, R.M.; Fuziah, M.Z.; Hong, J.Y.H.; Abqariyah, Y.; Mokhtar, A.H.; Wan Mohamud, W.N. TG : HDL-C Ratio Is a Good Marker to Identify Children Affected by Obesity with Increased Cardiometabolic Risk and Insulin Resistance. *Int. J. Endocrinol.* **2019**, *2019*, 8586167, doi:10.1155/2019/8586167.
6. Pediatric Test Reference Values—Mayo Clinic Laboratories. Available online: https://www.mayocliniclabs.com/test-info/pediatric/refvalues/ (accessed on 30 September 2020).
7. De Henauw, S.; Michels, N.; Vyncke, K.; Hebestreit, A.; Russo, P.; Intemann, T.; Peplies, J.; Fraterman, A.; Eiben, G.; de Lorgeril, M.; et al. Blood lipids among young children in Europe: Results from the European IDEFICS study. *Int. J. Obes.* **2014**, *38* (Suppl. S2), S67–S75, doi:10.1038/ijo.2014.137.
8. Kamoda, T.; Saitoh, H.; Inudoh, M.; Miyazaki, K.; Matsui, A. The serum levels of proinsulin and their relationship with IGFBP-1 in obese children. *Diabetes Obes. Metab.* **2006**, *8*, 192–196, doi:10.1111/j.1463-1326.2005.00479.x.
9. Schlenz, H.; Intemann, T.; Wolters, M.; González-Gil, E.M.; Nappo, A.; Fraterman, A.; Veidebaum, T.; Molnar, D.; Tornaritis, M.; Sioen, I.; et al. C-reactive protein reference percentiles among pre-adolescent children in Europe based on the IDEFICS study population. *Int. J. Obes.* **2014**, *38* (Suppl. S2), S26–S31, doi:10.1038/ijo.2014.132.
10. Lausten-Thomsen, U.; Christiansen, M.; Hedley, P.L.; Nielsen, T.R.H.; Fonvig, C.E.; Pedersen, O.; Hansen, T.; Holm, J.-C. Reference values for fasting serum resistin in healthy children and adolescents. *Clin. Chim. Acta* **2017**, *469*, 161–165, doi:10.1016/j.cca.2017.04.002.
